# Barriers to Conducting Clinical Trials in Developing Countries

**DOI:** 10.31486/toj.19.0068

**Published:** 2019

**Authors:** Adeel Khoja, Fizzah Kazim, Naureen Akber Ali

**Affiliations:** ^1^ Department of Medicine, Aga Khan University, Karachi, Pakistan; ^2^ Dow Medical College, Dow University of Health Sciences, Karachi, Pakistan; ^3^ School of Nursing and Midwifery, Aga Khan University, Karachi, Pakistan

## TO THE EDITOR

Clinical trials play a crucial role in determining the efficacy, safety, and adverse effects of new treatment methods before they are introduced to the general population.^1^ The double burden of disease in developing countries emphasizes the strong need to conduct clinical research to identify and implement cost-effective and novel treatment plans^2^ that provide guidelines for healthcare professionals working in environments with limited medical and surgical resources.^3^ A 2018 study revealed a staggering difference in clinical trial sites; approximately 83% of trials had been conducted in 25 high-income countries, whereas <5% had been conducted in 91 lower-middle or low-income countries (LMICs).^4^ Despite the high prevalence of disease in LMICs, substantial funding and research to address this gap are not available in most countries.

Key barriers that impede progress in clinical research are the lack of financial resources, the lack of skilled personnel, and regulatory and administrative issues.^5^

Alemayehu et al reported lack of funding as a common obstacle and noted that the majority of funding for clinical trials comes from western countries or pharmaceutical companies established in the West.^5^ In developing countries, governments allocate meager funding for research and overall health.

The lack of focus on clinical trials research in the curricula in medical schools and teaching hospitals^5^ and the lack of research-based higher educational institutions have led to a dearth of skilled personnel.^5^ Individuals with specialized training or experience in clinical trials often prefer to work abroad because of the greater opportunities, resulting in brain drain in their countries.^5^

Unnecessary delays in ethical approval procedures and complex and unreasonably strict government regulatory systems have further impeded progress.^6^ Additionally, certain cultural and religious beliefs that create fear of exploitation in the general population have also hampered advancement.^6^

Despite these challenges, LMICs showed the highest annual growth rate in clinical trials in the period 2006 to 2012 (14.7%) compared to the United States, high-income countries, upper-middle income countries, and low-income countries.^4^

Global collaboration among developed countries and LMICs is essential to foster clinical trial research. Clinical trials following ethical guidelines that cater to the health needs of people living in LMICs are needed. Significant investment is required in research infrastructure and research-based higher education centers. Governments need to implement changes that reduce approval times and speed regulatory processes to attract more funding for clinical trials.

## Notes

**Erratum** Boys JA, Bowdish ME, Subramanyan RK, Shemanski KA, Dhillon GS, Toda K, Perrillo J, Seoane L, Bates MJ, Parrino PE, Kooperkamp H, Valentine VG, Emory WB, Ochsner JL, McFadden PM. Fortification of preservation solution with nitroprusside does not alter lung allograft survival in clinical human lung transplantation. *Ochsner J*. 2019 Fall;19(3):235-240. doi: 10.31486/toj.19.0027. Erratum submitted by authors. Author name corrected. The name Ram Kumar Subramanyan as originally published is corrected to S. Ram Kumar.

## Notes

**Erratum** Mobley D, Baum NH, Beattie A, Nemeroff C. When imagination becomes a disease: dealing with hypochondriacal patients in clinical practice. Ochsner J. 2019 Summer;19(2):70-73. doi: 10.31486/toj.18.0123. Erratum submitted by authors. Author affiliation corrected. The affiliation Baylor College of Medicine, Houston, TX as originally published for David Mobley, MD is corrected to Associate Professor, Clinical Urology, Weill-Cornell Medicine, New York, NY.
